# Risk factors for post-stroke spasticity: a retrospective study

**DOI:** 10.3389/fneur.2024.1478206

**Published:** 2024-12-17

**Authors:** Chuanxi Zhu, Lingxu Li, Long Qiu, Guangcheng Ji

**Affiliations:** ^1^Department of Rehabilitation Medicine, Changchun University of Chinese Medicine, Changchun, Jilin, China; ^2^Tongliao City Hospital, Tongliao, Inner Mongolia, China; ^3^Encephalopathy Center, The Third Affiliated Clinical Hospital of Changchun University of Chinese Medicine, Changchun, Jilin, China; ^4^Changchun University of Chinese Medicine, Changchun, Jilin, China

**Keywords:** stroke, spasticity, influence factors, post-stroke spasticity, retrospective study

## Abstract

**Background:**

Post-stroke spasticity (PSS) is a common complication after stroke and is an important cause of high rates of disability after stroke. At present, modern medicine has made great progress in the treatment of PSS, ‘early detection, early treatment’ has become a general consensus for the treatment of PSS in the clinic. Clarifying the risk factors of PSS can help to detect and treat the functional disorders caused by PSS at an earlier stage.

**Methods:**

This is a retrospective study. 436 stroke patients who visited the Neurology Department of the Third Affiliated Clinical Hospital of Changchun University of Chinese Medicine from June 2020 to November 2020 were selected as study subjects, and finally 257 patients were included in the final analysis, and divided into 101 cases with spasticity and 156 cases without spasticity, depending on whether or not the stroke victim had a spasm at the time of admission.

**Results:**

The multivariate regression analysis showed that basal ganglia as the cerebral hemorrhage or infarction site (OR = 4.930, 95%CI = 2.743–8.86, *p* = 0.000), cerebral hemorrhage or infarction volume (OR = 1.087, 95%CI = 1.016–1.164, *p* = 0.016) and NIHSS scores (OR = 1.232, 95%CI = 1.089–1.393, *p* = 0.001) are independent influencing factors and independent risk factors for spasticity (*p* < 0.05). A risk prediction model for spasticity in stroke patients is derived with the multivariate logistic regression analysis Logit (P) = 1.595 * Basal ganglia +0.084 * infarct volume + 0.208 * NIHSS scores – 2.092. An evaluation of the goodness of fit using the ROC curve showed AUC (95% CI) = 0.786 (0.730–0.843), an indication of a high degree of model fit.

**Conclusion:**

Independent risk factors for Post-stroke spasticity include basal ganglia as the cerebral hemorrhage or infarction site, cerebral hemorrhage or infarction volume and NIHSS scores.

## 1 Introduction

Cerebral apoplexy, or stroke, is the third leading cause of disability in adults and the second leading cause of deaths globally (1, 2). Post-stroke spasticity (PSS) is a form of increased muscle tone where pathological changes in the upper motor neurons lead to impaired sensory and motor controls. It is a motor disorder characterized by a velocity-dependent increase in tonic stretch reflexes with tendon hyperreflexia resulting in abnormal postures and movement patterns in stroke patients. It is a major contributing factor to high post-stroke disability rates (3–5). Studies have found the treatment cost to be higher in stroke patients with PSS than those without PSS (6). The pathogenesis of PSS is complex and various researchers have proposed different ideas and definitions (7–9). At present, modern medicine has made great progress in the treatment of PSS, including botulinum toxin injections, intrathecal baclofen pumps, etc., and “early detection, early treatment” has become a general consensus for the treatment of PSS in the clinic (10, 11). This study was analyzed from the perspective of prevention. Through the investigation and study of the relevant samples, the study aims to understand the incidence of spasticity after stroke, screen the relevant risk factors of spasticity and construct a risk prediction model, which will further provide a reliable theoretical basis for exploring the early rehabilitation therapies, reducing the incidence of spasticity and slowing down the degree of spasticity. Therefore, this study investigated and studied the relevant samples to understand the incidence of PSS, and screened the relevant risk factors of spasticity to provide additional reliable theoretical basis for the effective prevention of PSS in clinical practice.

## 2 Methods

**Figure 1:**
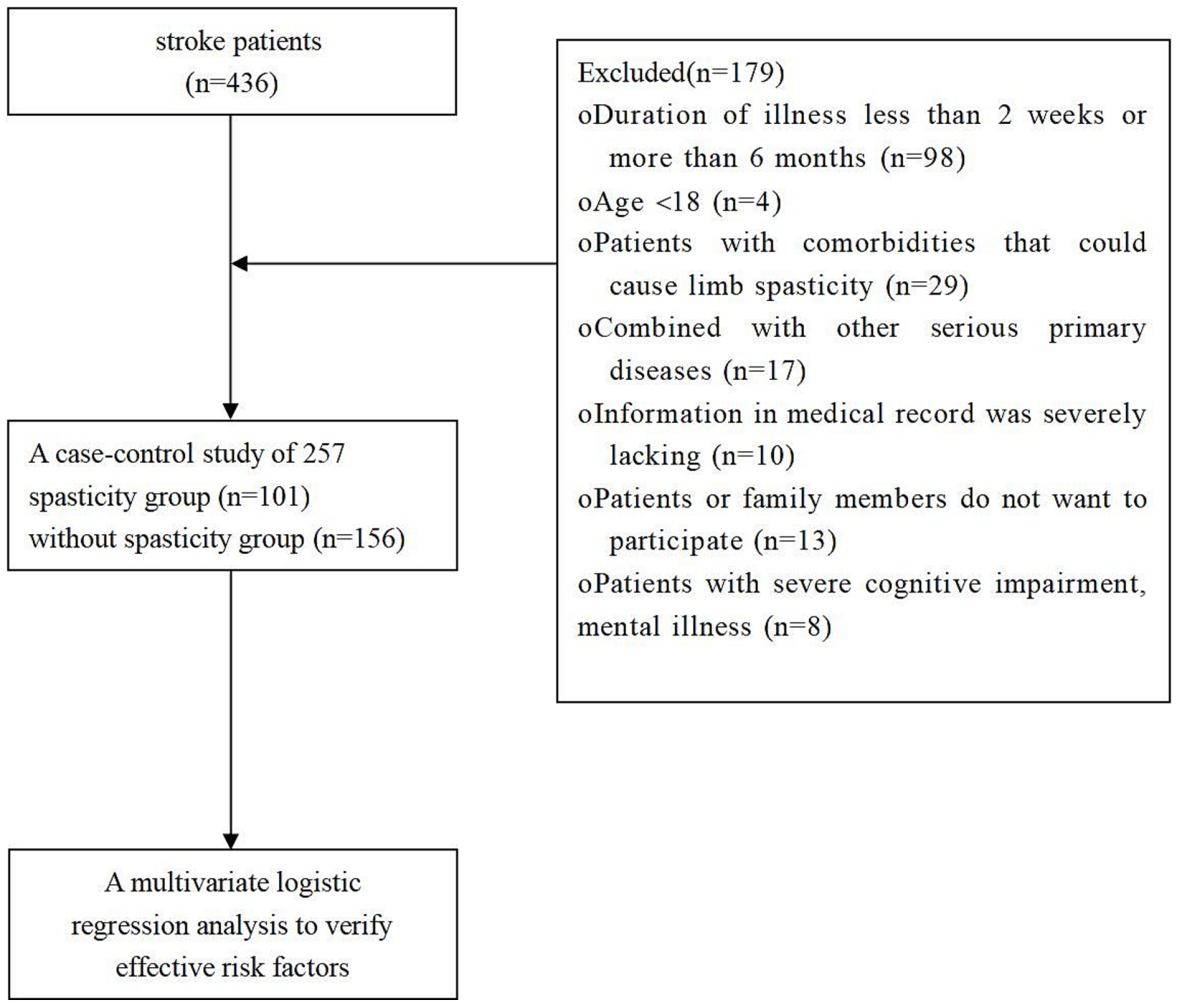
The flow chart of the retrospective study.

This is a retrospective study. A total of 436 stroke patients who visited the Neurology Department of the Third Affiliated Clinical Hospital of Changchun University of Chinese Medicine from June 2020 to November 2020 were selected as study subjects, and finally 257 patients were included in the final analysis, and divided into 101 cases with spasticity and 156 cases without spasticity, depending on whether the individual patient experienced spasticity in the 6 months after the stroke (Any muscle considered to be in spasticity if a value of 1 or more in any muscle in the Modified Ashworth Scale) (Figure 1). From the electronic database of medical records, the investigators recorded information such as the age and gender of study subjects, their medical history in terms of smoking, drinking, hypertension, diabetes, and hyperlipidemia, their dominant hands (left or right hand), as well as observed and recorded the cerebral hemorrhage or infarction by side (left or right), the site of cerebral hemorrhage or infarction (frontal lobe, parietal lobe, temporal lobe, occipital lobe, thalamus, hippocampus, basal ganglia, cerebellum, midbrain, etc.). The volume of the cerebral hemorrhage or infarction was derived from MRI examinations and FLAIR images were used to measure the size of the focal area. Post-processing software was applied on the disclosed relevant sequence to outline the contours of the cerebral hemorrhage or infarction at each layer of the cerebral hemorrhage or infarction in order to automatically calculate the area, these areas were then added layer by layer, and finally multiplied by the layer thickness and inter-layer spacing to obtain the cerebral hemorrhage or infarction volume. Neurological deficit scores (NIHSS scores) and modified Ashworth scores assessed on admission in patients’ e-cases were collected. The data were summarized in a database of observation tables using Excel, then validated and checked for errors before statistical analyses were performed.

### 2.1 Diagnostic criteria

Based on the diagnostic criteria for cerebral infarction and cerebral hemorrhage in the Chinese Guidelines for Diagnosis and Treatment of Acute Ischemic Stroke 2018 (12) and the Chinese Guidelines for Diagnosis and Treatment of Subarachnoid Hemorrhage 2019 (13). All cases were confirmed by cranial CT or MRI. The modified Ashworth method was used to evaluate the severity of limb spasticity. A final diagnosis was then carried out to confirm if there was limb spasticity.

### 2.2 Inclusion criteria

(1) Met the diagnostic criteria for cerebral hemorrhage or infarction; (2) Course of disease was between 2 weeks to 6 months; (3) Age ≥ 18; (4) Consciousness and stable vital signs. (5) Patients or family members gave informed consent and participated voluntarily.

### 2.3 Exclusion criteria

(1) Patients with comorbidities that could cause limb spasticity; (2) Individuals with severe primary diseases such as impairment of liver, kidney, hematopoietic system, and endocrine system; (3) Individuals with severe cognitive impairment, mental illness; (4) Pregnant and lactating women; (5) Information in medical record was severely lacking.

### 2.4 Sample size estimation method

The sample size for this trial was estimated by calculating the required sample size based on logistic regression analysis in Medical Statistics, Second Edition (14): requires a minimum sample size of more than 10 times the number of independent variables, so as to reflect more realistically the relationship between the independent variable and the dependent variable.

Sample size = number of independent variables × 10.

In the formula: the number of independent variables is 20.

Therefore, it was calculated that at least 200 were required for this survey. The total number of cases finally collected in this study was 257 which fulfils the requirement of study design.

### 2.5 Data processing

Statistical analyses of the data were performed using SPSS 27.0. Measurement data with a normal distribution was presented as _x   ± s while data with a non-normal distribution was presented as M (IQR), and the Mann–Whitney U test was used to compare between the groups. Count data was represented by n(%), and the chi-square test or Fisher’s exact test was used for comparisons between the groups. Logistic regression was used to identify the factors that affect PSS. The difference is considered statistically significant if *p* < 0.05.

## 3 Results

As seen in Table 1, comparisons between the groups showed that the differences in involvement of basal ganglia, cerebral hemorrhage or infarction volume, and NIHSS scores to be statistically significant (*p* < 0.05), an indication that these may be factors that affect spasticity. However, the impact of confounding factors for spasticity was not accounted for. Therefore, a multivariate regression analysis that took into account the effects of confounding factors was carried out to identify independent factors that affect spasticity.

**Table 1:** Comparison of various indicators in the groups with and without spasticity.

	No spasticity (*n* = 156)	With spasticity (*n* = 101)	*X^2^/z*	*P*
Age (years)	64 (56,69)	62 (55,69)	−0.589	0.556
Gender (%)			1.522	0.217
Male	98 (62.82)	71 (70.30)		
Female	58 (37.18)	30 (29.70)		
Smoking history (%)	39 (25.00)	30 (29.70)	0.690	0.406
Alcohol history (%)	34 (21.79)	23 (22.77)	0.034	0.854
Hypertension history (%)	109 (69.87)	76 (75.25)	0.879	0.349
Diabetes history (%)	51 (32.69)	30 (29.70)	0.254	0.614
Hyperlipidemia history (%)	14 (8.97)	10 (9.90)	0.062	0.803
Dominant hand (%)			0.348	0.555
Left	3 (1.92)	1 (0.99)		
Right	153 (98.08)	100 (99.01)		
Hemorrhage/Infracted side (%)			0.013	0.909
Left	73 (46.79)	48 (47.52)		
Right	83 (53.21)	53 (52.48)		
Hemorrhage/Infract site (%)				
Frontal lobe (%)	36 (23.08)	22 (21.78)	0.059	0.808
Parietal lobe (%)	38 (24.36)	22 (21.78)	0.227	0.633
Temporal lobe (%)	26 (16.67)	19 (18.81)	0.195	0.659
Occipital lobe (%)	13 (8.33)	12 (11.88)	0.879	0.349
Thalamus (%)	28 (17.95)	19 (18.81)	0.031	0.861
Hippocampus (%)	7 (4.49)	2 (1.98)	0.519	0.471
Basal ganglia (%)	31 (19.87)	59 (58.42)	40.025	0.000*
Cerebellum (%)	20 (12.82)	13 (12.87)	0.000	0.991
Midbrain (%)	15 (9.62)	10 (9.9)	0.006	0.940
Hemorrhage/Infarct volume (cm^3^)	212.3 (75.39,1257.32)	1949.1 (877.2,4252.5)	−8.202	0.000*
NIHSS scores	3 (2,5)	4 (2,8)	−4.341	0.000*

*p < 0.05.

**Figure 2:**
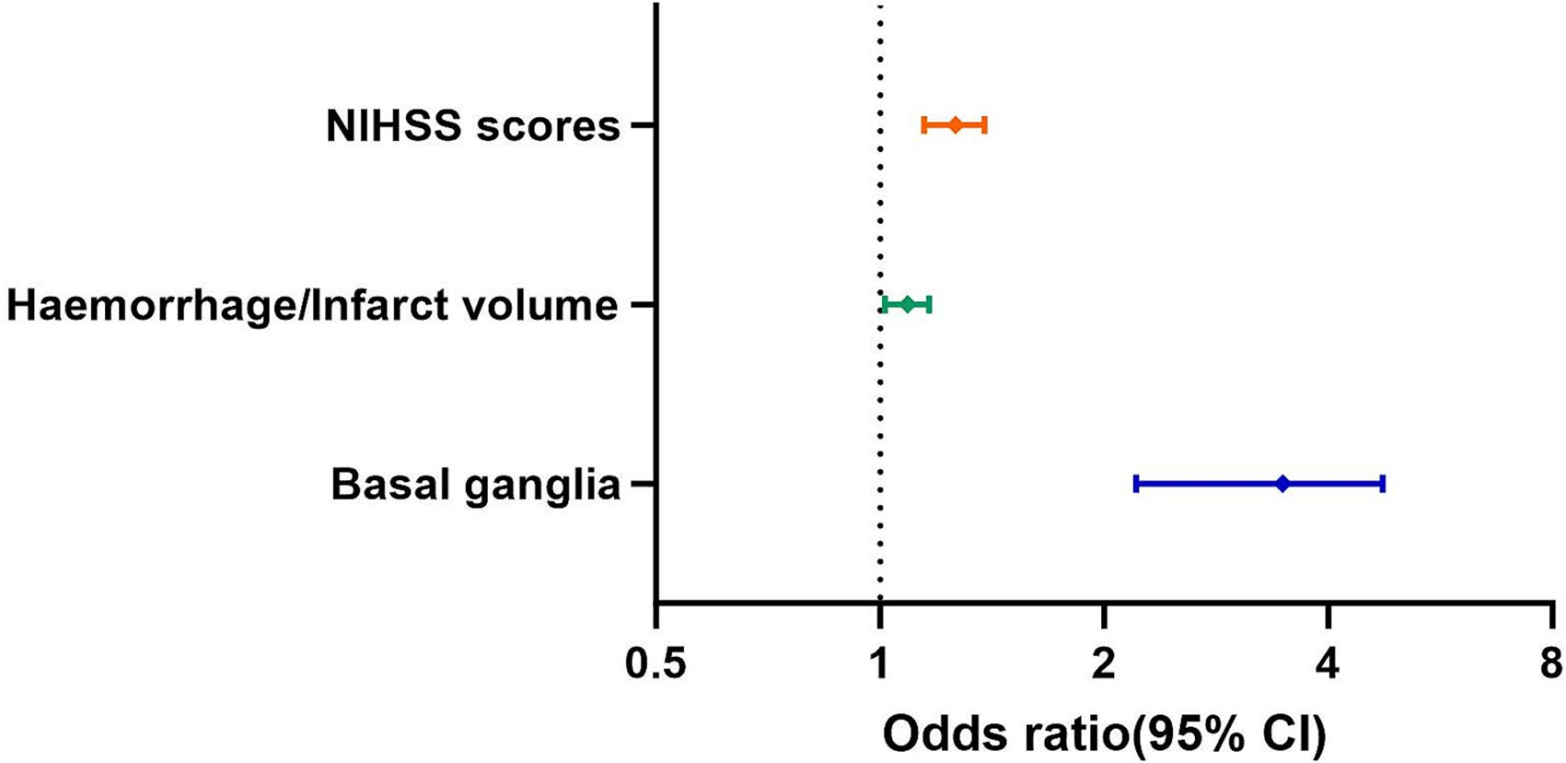
Risk factors for post-stroke spasticity- forest map.

As seen in Table 2, results from the multivariate regression analysis showed that basal ganglia as the cerebral hemorrhage or infarction site, cerebral hemorrhage or infarction volume and NIHSS scores are independent influencing factors and independent risk factors for spasticity (*p* < 0.05) (Figure 2). Specifically, spasticity is more likely to occur when the cerebral hemorrhage or infarction site is the basal ganglia, the larger the area of cerebral hemorrhage or infarction the more likely it is to lead to spasticity, while a higher NIHSS scores indicates a higher probability of spasticity. All other indicators are not independent influencing factors for spasticity.

**Table 2:** Multivariate regression analysis.

	B	S.E.	Wald	*P*	OR	95% CI	
						Lower	Upper
Basal ganglia	1.595	0.299	28.452	0.000*	4.930	2.743	8.86
Hemorrhage/Infarct volume	0.084	0.035	5.779	0.016*	1.087	1.016	1.164
NIHSS scores	0.208	0.063	10.966	0.001*	1.232	1.089	1.393
Constant	−2.092	0.315	44.242				

*p < 0.05.

**Figure 3:**
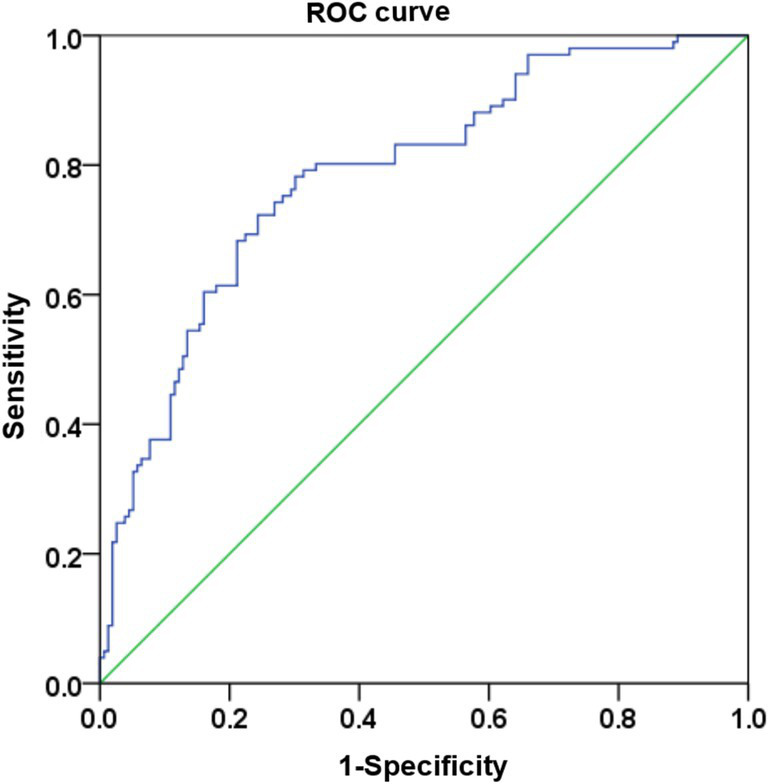
ROC curve.

A risk prediction model for spasticity in stroke patients is derived with the multivariate logistic regression analysis: Logit (P) = 1.595 * Basal ganglia +0.084 * infarct volume + 0.208 * NIHSS scores – 2.092. The Hosmer-Lemeshow test results in Table 3 are *X^2^* = 13.828, and *p* = 0.086, which means that there is no significant difference between the predicted value and the actual value in the Hosmer-Lemeshow test. An evaluation of the goodness of fit using the ROC curve showed AUC (95% CI) = 0.786 (0.730–0.843), an indication of a high degree of model fit (Figure 3).

**Table 3:** Hosmer-Lemeshow test for goodness of fit.

*X^2^*	Degrees of freedom	Significance
13.828	8	0.086

## 4 Discussion

Spasticity is a common post-stroke complication, and approximately one-third of stroke patients will experience spasticity within 3 months of onset of stroke (15–17). Spasticity is harmful to stroke patients, requiring them to undergo long-term rehabilitation and causing a series of physical and psychological problems that seriously affect their motor function and daily living activities (18, 19). Studies have shown that patients with PSS often suffer from psychological problems such as depression and anxiety, cognitive impairment such as memory loss, and poor concentration (20–24). Post-spasm pain can also lead to sleep disorders. All these seriously affect the patient’s quality of life.

In recent years, as modern medical science and technology develops, more clinical treatments for PSS have emerged, such as oral antispasmodics, botulinum toxin injections, physiotherapy, antispastic positioning, as well as acupuncture, moxibustion and traditional Chinese medicine (25–31). Currently, the most rapid and effective western medical treatments for spasticity are oral anti-spasmodic drugs and local injection of botulinum toxin (32, 33). The early stage of PSS has also achieved better clinical outcomes through antispastic positioning (25). Acupuncture and moxibustion, massage (tuina) and traditional Chinese medicine have the advantages of simplicity and speed, and have achieved remarkable efficacy in the clinical treatment of PSS (34). If the point of treatment is too late, resulting in abnormal movement patterns and postures that have developed, the only way to treat it is through surgery, which is effective but has the disadvantages of a high coefficient of difficulty and high treatment costs (35, 36). Therefore, early detection of spasticity and carrying out effective and rapid treatment are currently the focus of clinical treatment of PSS, which not only reduces related complications, but also shortens the treatment cycle and reduces the burden on the patient’s family. Clarifying the risk factors of PSS can help to detect and treat the functional disorders caused by PSS at an earlier stage, improve the rehabilitation efficacy of the patients, and enhance their ability to return to their families and society.

There are many risk factors for PSS (37–39). NIHSS scores is an important indicator for assessing post-stroke neurological damage. A higher NIHSS scores means a more severe decline in the patient’s neurological functions (40, 41). Studies have shown that PSS patients have relatively higher NIHSS scores (15). This study found the NIHSS scores to be significantly higher in the group with PSS when compared to the group with no PSS. There is a significant correlation between the incidence of PSS and NIHSS scores (*p* < 0.05). In the multivariate analysis, NIHSS scores is an independent risk factor for PSS (OR = 1.515), and consistent with the findings of Ryu et al. (42). Relevant studies have also found (42) NIHSS scores to be a significant predictor of the occurrence of PSS. Basal ganglia refers to a group of nerve nuclei located deep in the brain, and comprises of the striatum, caudate nucleus, and globus pallidus. These sub-components play key roles in motor, emotional, cognitive, and focus. A damaged basal ganglia can lead to symptoms like muscle tone disorders, and spasticity (43, 44). Studies have demonstrated a close relationship between the site of brain injury and the occurrence of spasticity (45). This study found the proportion of basal ganglia injury to be higher in patients with PSS than those without PSS. There was a significant correlation between the incidence of PSS and basal ganglia injury (*p* < 0.05). Based on the relevant multivariate analysis, basal ganglia injury is an independent influencing factor for the incidence of PSS (OR = 6.693). Studies worldwide have also confirmed (46, 47) that patients with basal ganglia injury have the highest risk of PSS. The size of cerebral hemorrhage or infarction also has a correlation with the occurrence of PSS, and this study showed that there was a significant difference in the comparison of the size of cerebral hemorrhage or infarction between patients with spasticity after stroke and those without spasticity (*p* < 0.05), suggesting that large cerebral hemorrhage or infarction may be one of the factors influencing the development of limb spasticity after stroke. Related studies have shown that patients with less spasticity after stroke have smaller areas of cerebral hemorrhage or infarction, while the opposite is true for patients with severe spasticity (47, 48).

Some studies have found that the incidence of spasticity is higher in hemorrhagic strokes than in ischemic strokes, which may be related to the fact that hemorrhagic strokes have a higher degree of disability (49). Hemorrhagic stroke and ischemic stroke have very different pathological mechanisms. In addition to early local cerebral hemorrhage, hemorrhagic stroke is accompanied by a variety of pathological changes in the brain tissue in the hemorrhage area, such as ischemia, hypoxia, inflammatory response, neuronal degeneration, necrosis and apoptosis (50). Hemorrhagic stroke and ischemic stroke have different degrees and extent of damage to the central nervous system, and the onset of spasticity in different stroke types was not further analyzed in this study, the effects of cerebral hemorrhage and cerebral ischemia on spasticity will be further specifically analyzed in future studies. Other researchers have found that stroke patients with a history of previous stroke, that is, patients who are not the first stroke, have a higher proportion of spasticity, and the reason is related to the aggravation of brain tissue damage after a second stroke, and further neurological function damage leads to an increased likelihood of spasticity (51). It is now generally accepted that the incidence of spasticity is relatively low in the acute phase of stroke, and the incidence will gradually increase as the stroke course progresses and lengthens (52). Therefore, in this study, patients in the recovery period after stroke were selected as the research subjects to further clarify the importance of prevention of spasticity after stroke and early rehabilitation intervention on the recovery of neurological function after stroke.

This study retrospectively analyzed data on the patient’s conditions and used multivariate logistic regression to identify factors that may influence spasticity, including the involvement of basal ganglia, cerebral hemorrhage or infarction volume, and NIHSS scores. Independent influencing factors for spasticity (*p* < 0.05) include basal ganglia as the cerebral hemorrhage or infarction site, cerebral hemorrhage or infarction volume, and NIHSS scores. Due to the limited time and funding, this study has shortcomings in areas like study design and methods, and the results cannot comprehensively encompass all risk factors for PSS. Furthermore, this study adopts a retrospective approach, and is not able to dynamically track and observe the development of spasticity. In selecting the sample, only patients from a single center were selected for the study, so the generalization of the results to patients in other geographical regions should be approached with caution. In future research, a multicenter study with a larger sample size and longer follow-up duration will be conducted for a more comprehensive and in-depth investigation on the risk factors of PSS. A more scientific study plan will be adopted to provide a better scientific basis with regards to the prevention of PSS.

## 5 Conclusion

Independent risk factors for Post-stroke spasticity include basal ganglia as the cerebral hemorrhage or infarction site, cerebral hemorrhage or infarction volume and NIHSS scores.

## Data Availability

The original contributions presented in the study are included in the article/supplementary material, further inquiries can be directed to the corresponding author.
